# Lepromatous Leprosy Presenting With Preserved Sensation: A Case Report

**DOI:** 10.7759/cureus.109659

**Published:** 2026-05-25

**Authors:** Akinlolu C Shiyanbola, Paulinus C Emezie, Olanrewaju Saheed, Olagoke Erinomo, Oladipo Omoseebi

**Affiliations:** 1 Internal Medicine/Dermatology, Federal Teaching Hospital, Ido Ekiti, NGA; 2 Department of Medicine, College of Health Sciences, Afe Babalola University, Ado Ekiti, NGA; 3 Internal Medicine/Nephrology, Federal Teaching Hospital, Ido Ekiti, NGA; 4 Internal Medicine/Pulmonology, Federal Teaching Hospital, Ido Ekiti, NGA; 5 Anatomic Pathology, Federal Teaching Hospital, Ido Ekiti, NGA

**Keywords:** lepromatous, leprosy, mdt, multi drug treatment, timber, tuberculoid

## Abstract

Leprosy, which is also called Hansen's disease, is a chronic infectious disease that predominantly affects the skin and the nerves. It is caused by *Mycobacterium leprae*. The skin, peripheral nerves, upper respiratory tract mucous surface, and the eyes are mostly affected during the course of the disease. Leprosy can occur at any age, from early infancy to very old age. This case report describes a 43-year-old Nigerian man who works as a timber contractor. He presented with a month's history of the eruption of non-itchy papules first noticed on the face and progressively worsened till the patient had infiltration of the facial skin with leonine facie. Similar papules and nodules were noted in other parts of the body. The nodules on the lower limbs were ulcerated and septic. The earlobes were also infiltrated with oil-drop nodules. There was a history of weight loss, noticed at the same time as the lesions. Skin biopsy was done for histology and immunohistochemistry; results confirmed the diagnosis of lepromatous leprosy. Of relevance in this case report is the fact that light touch sensation was preserved in all the affected parts of the body at the initial presentation. Following diagnosis, our patient was counseled and was subsequently commenced on multi-drug therapy comprising rifampicin, dapsone, and clofazimine, with clinical improvement

## Introduction

Mycobacterium leprae is an intracellular parasitic pathogen that cannot be cultivated in artificial media and multiplies very slowly, with optimal growth at approximately 30°C; consequently, it prefers cooler areas of the human body [1]. Scalp, axilla, and groin are relatively less commonly affected due to their higher temperature, although involvement may occur in advanced disease. Nigeria remains among the countries with a relatively high burden of leprosy, reporting approximately 1,000-10,000 new cases annually according to the Centers for Disease Control and Prevention [2]. 

During the initial stages of leprosy, small sensory, autonomic, and motor nerve fibers in the skin are damaged. The damaged nerves usually result in hair loss in the area, a loss of the ability to sweat, and numbness [1]. As the disease progresses, more damage to the nerves may result in skin dryness, more numbness, and muscle weakness or paralysis in the affected areas. Leprosy is diagnosed clinically based on at least one cardinal sign, including definite sensory loss in a hypopigmented or erythematous skin lesion, thickened peripheral nerves with sensory or motor impairment, or the presence of acid-fast bacilli on slit-skin smear [3]. Our patient in this case report, a 43-year-old Nigerian man, presented with skin lesions consistent with leprosy, however with preserved sensation. The preserved sensation put the diagnosis in doubt, necessitating a skin biopsy for confirmation. Following the diagnosis, our patient was counseled and was subsequently commenced on a multi-drug therapy (MDT) regimen comprising rifampicin, dapsone, and clofazimine, with clinical improvement.

## Case presentation

On the 14th of October 2021, the Dermatology unit of the Federal Teaching Hospital Ido-Ekiti reviewed a 43-year-old man who is a timber contractor, a Nigerian who was born in Nigeria and resides in Nigeria.

**Figure 1:**
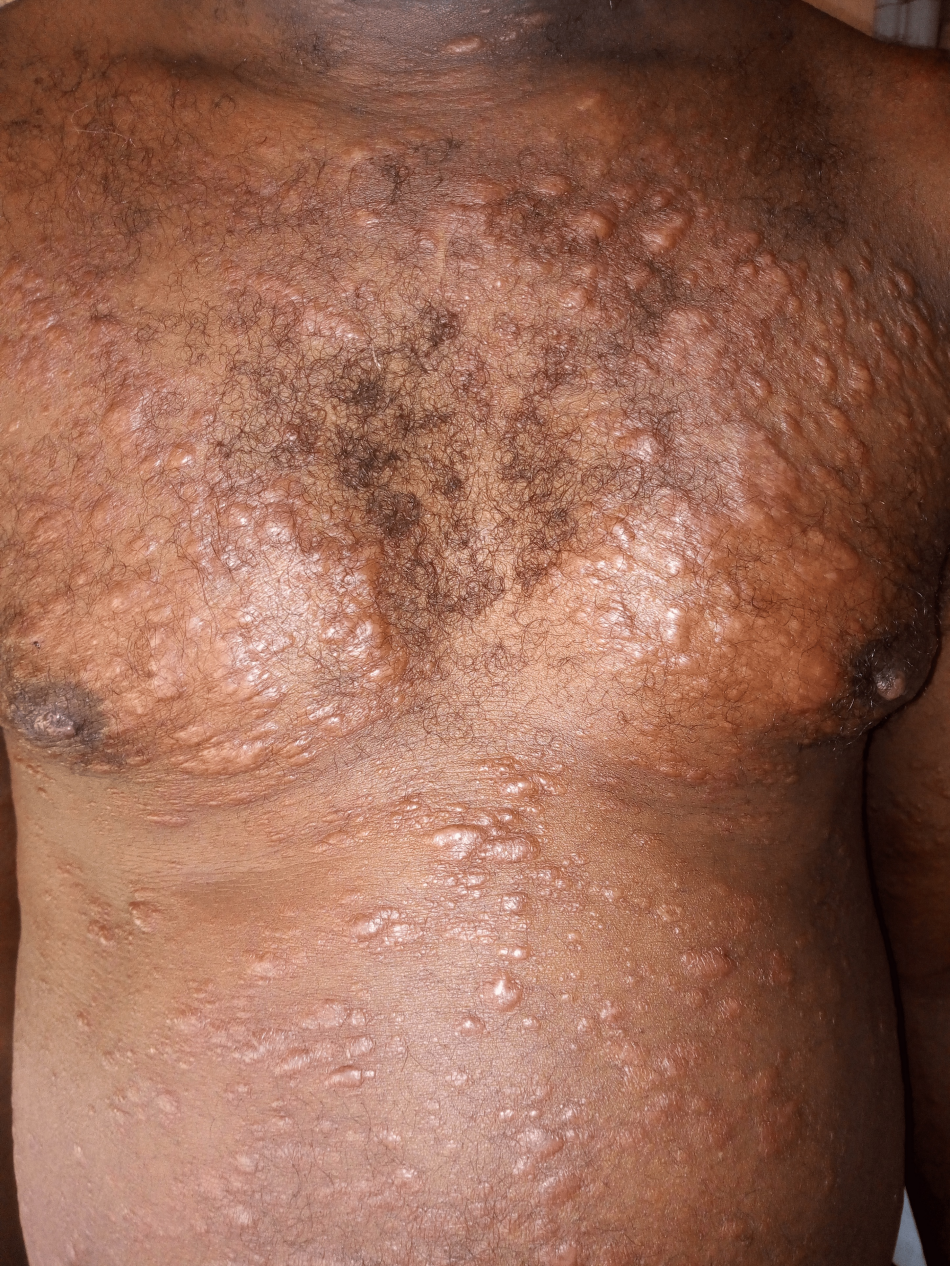
Multiple non-pruritic papules and nodules involving the face, showing diffuse infiltration and early leonine facies prior to treatment in lepromatous leprosy.

**Figure 2:**
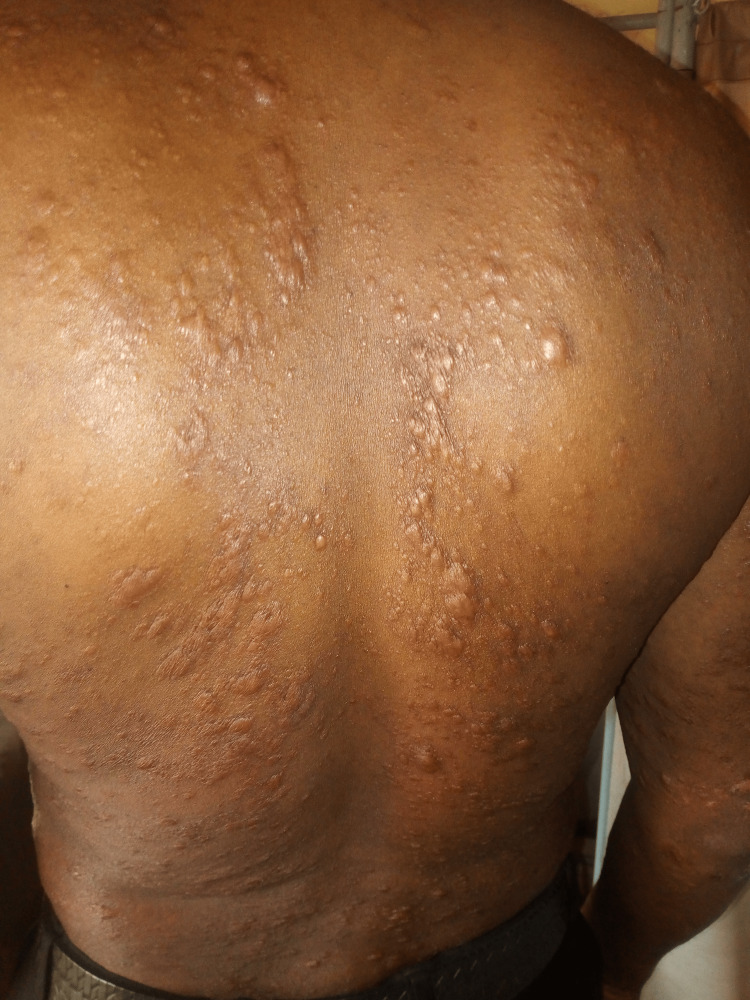
Multiple non-pruritic papules and nodules showing diffuse infiltration prior to treatment in lepromatous leprosy.

He presented with a seven-month history of eruption of non-itchy papules first noticed on the face, which progressively worsened till the patient had infiltration of the facial skin with leonine facie. Similar papules and nodules were seen on the other parts of the body. The nodules on the lower limbs were ulcerated and septic. The earlobes were also infiltrated with oil-drop nodules. There was a history of weight loss, noticed at the same time as the lesions. There was no history of nose bleeding, and no painless resorption of any digits on the extremities. There was no known medical condition prior to onset of symptoms, and no history of contact with any person with similar lesions. Images of the papules are noted in Figures 1, 2. 

On skin examination, papular eruptions were seen covering most parts of the skin, with ulcerations and septic appearance of the lesions on the lower limbs. Light touch sensation was preserved in all the affected parts of the body. No digit was missing. Other systems examination and vital signs were satisfactory.

Due to the severity of the skin lesions with preserved light touch sensation, the diagnosis of leprosy (Hansen's disease) was not initially clear, and a differential of cutaneous T-cell lymphoma (CTCL) was considered.

The management plan was to do basic laboratory investigations and skin biopsy. He was vaccinated (anti-tetanus serum (ATS) and tetanus toxoid) and commenced on antibiotics. He was subsequently given a two-week appointment to be seen at the dermatology clinic.

He presented at the dermatology unit two weeks later with the results of his investigations, which are summarized in Table 1.

**Table 1:** Laboratory Investigations PCV: packed cell volume; WBC: white blood cell count; FBG: fasting blood glucose; ALT: alanine aminotransferase; AST: aspartate aminotransferase; ALP: alkaline phosphatase; Na: sodium; K: potassium; Cl: chloride; HCO₃⁻: bicarbonate; Cr: creatinine; eGFR: estimated glomerular filtration rate. Reference ranges were based on institutional laboratory standards.

Test	Result	Reference Range
Packed cell volume (PCV)	0.32	40–52% (men)
White blood cell count (WBC)	10.3 × 10⁹/L	4.0–11.0 × 10⁹/L
Neutrophils	0.5	40–70%
Lymphocytes	0.43	20–45%
Monocytes	0.07	2–10%
Platelet count	367 × 10⁹/L	150–450 × 10⁹/L
Hypochromia	++	Normal = absent
Fasting blood glucose (FBG)	5.6 mmol/L	3.9–5.5 mmol/L
2-hour postprandial glucose	6.6 mmol/L	<7.8 mmol/L
Total bilirubin	8.9 µmol/L	5–21 µmol/L
Conjugated bilirubin	6.9 µmol/L	0–5 µmol/L
Alanine aminotransferase (ALT)	45 U/L	10–50 U/L
Aspartate aminotransferase (AST)	49 U/L	10–50 U/L
Alkaline phosphatase (ALP)	54 U/L	44–147 U/L
Total protein	87 g/L	60–80 g/L
Albumin	33 g/L	35–50 g/L
Sodium (Na)	134 mmol/L	135–145 mmol/L
Potassium (K)	3.8 mmol/L	3.0–5.0 mmol/L
Chloride (Cl)	106 mmol/L	98–106 mmol/L
Bicarbonate (HCO₃⁻)	Not available	22–28 mmol/L
Urea	3.3 mmol/L	3–7 mmol/L
Creatinine (Cr)	84 µmol/L	60–115 µmol/L (men)
Estimated glomerular filtration rate (eGFR)	113 mL/min/1.73 m²	≥90 mL/min/1.73 m²

Laboratory investigations (Table 1) were largely within normal limits, except for features suggestive of anemia of chronic disease, with no significant abnormalities to suggest alternative systemic pathology. The management plan was to continue antibiotic cover while awaiting the result of the skin biopsy histology. The patient was also seen by the ENT unit, where he was evaluated for nasal polyp with active rhinosinusitis. 

**Figure 3:**
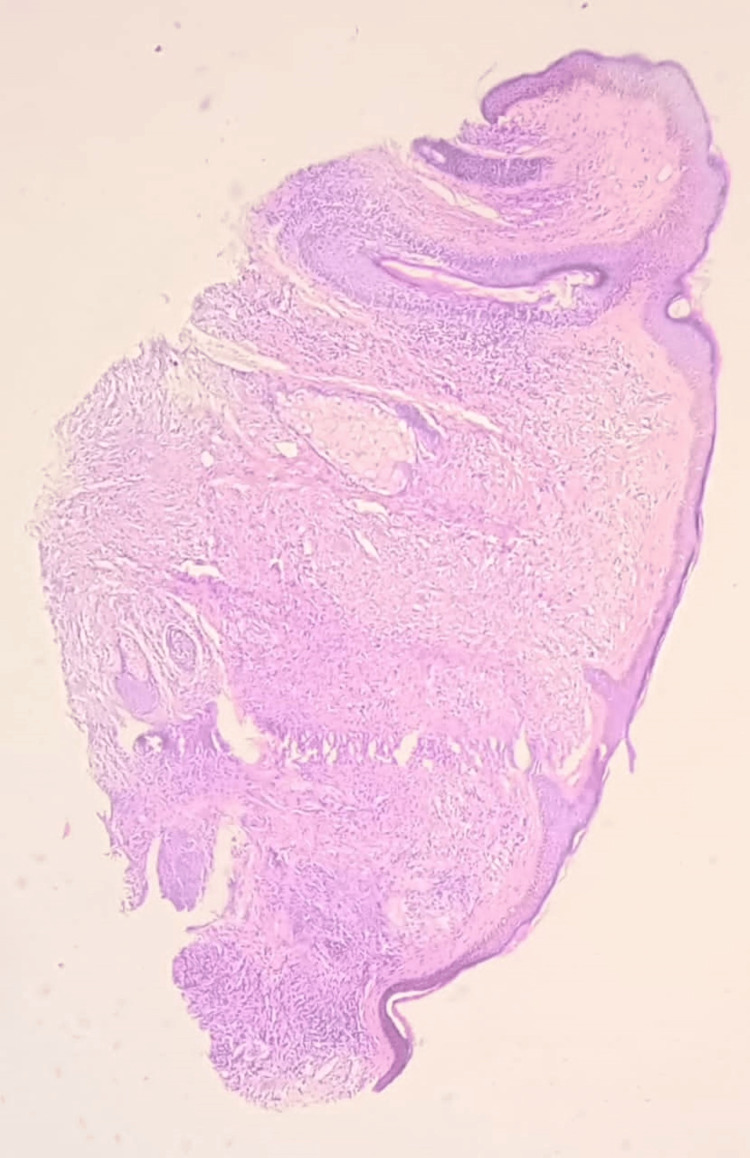
Histology (Hematoxylin and Eosin stain) showing diffuse dermal infiltration by sheets of histiocytes with perivascular lymphohistiocytic infiltrates, consistent with features of lepromatous leprosy.

On the 28th of October 2021, the histology result of the tissue sample was received (Figure 3).

**Figure 4:**
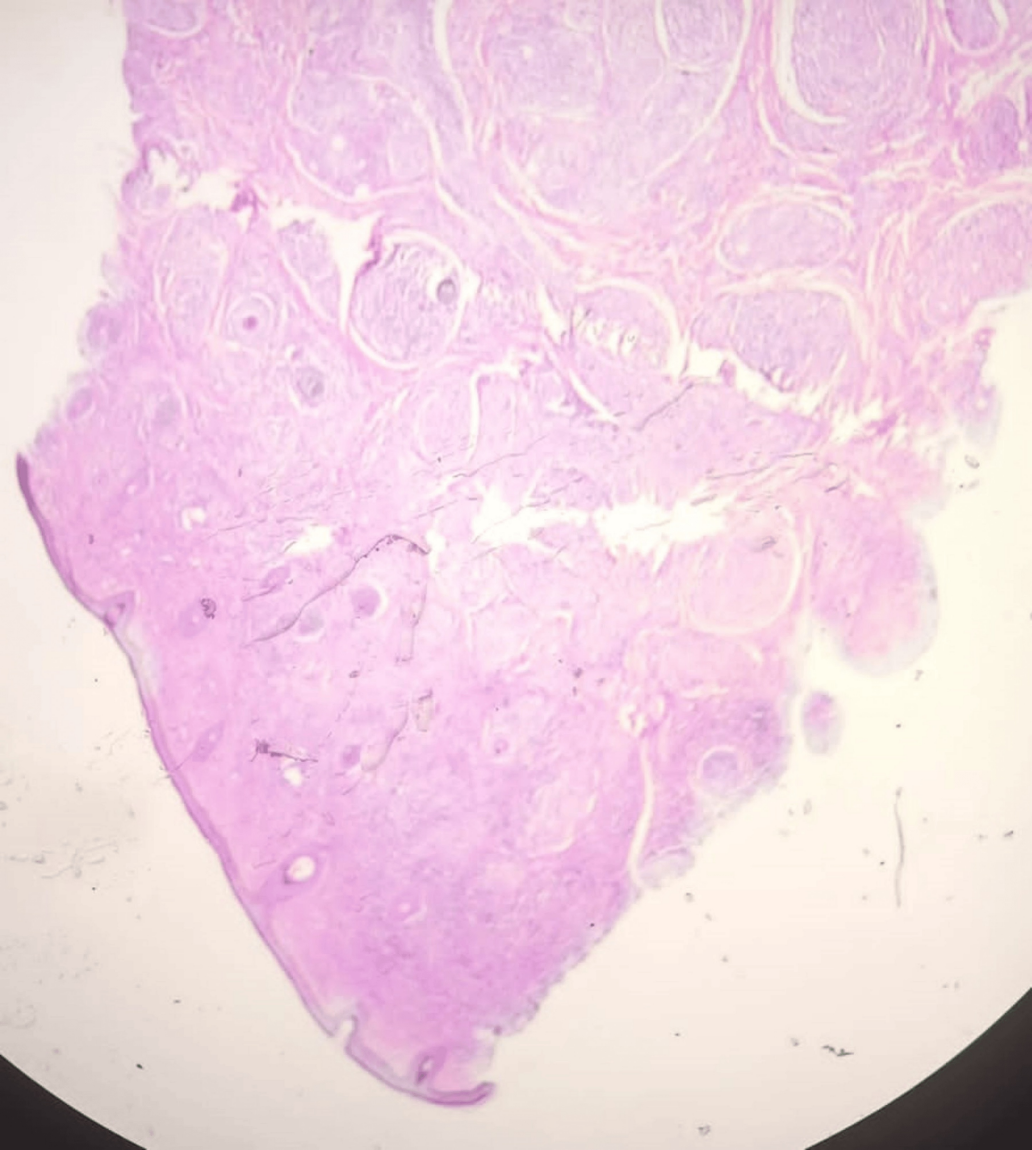
Histology (Hematoxylin and Eosin stain) showing parakeratosis with diffuse infiltration of the dermis by histiocytes arranged in poorly circumscribed aggregates, with associated perivascular lymphohistiocytic infiltrates, in keeping with lepromatous leprosy.

The above histology features were said to be suggestive of T-cell lymphoma initially. However, on the 5th of January 2022, part of the tissue sample that was stored and preserved was sent out again for immunohistochemical analysis and showed cells that were positive for CD3, CD45, and CD68, but negative for CD5 and CD20, with a microscopic description of mixed inflammatory cell infiltrates within the dermis. The cells were said to consist of histocytes, lymphocytes, and plasma cells. The inflammatory cells were said to tend to aggregate and infiltrate around nerve bundles. These new findings disputed the diagnosis of CTCL, warranting further histology analysis (Figure 4).

The above findings ruled out CTCL and confirmed lepromatous leprosy.

**Figure 5:**
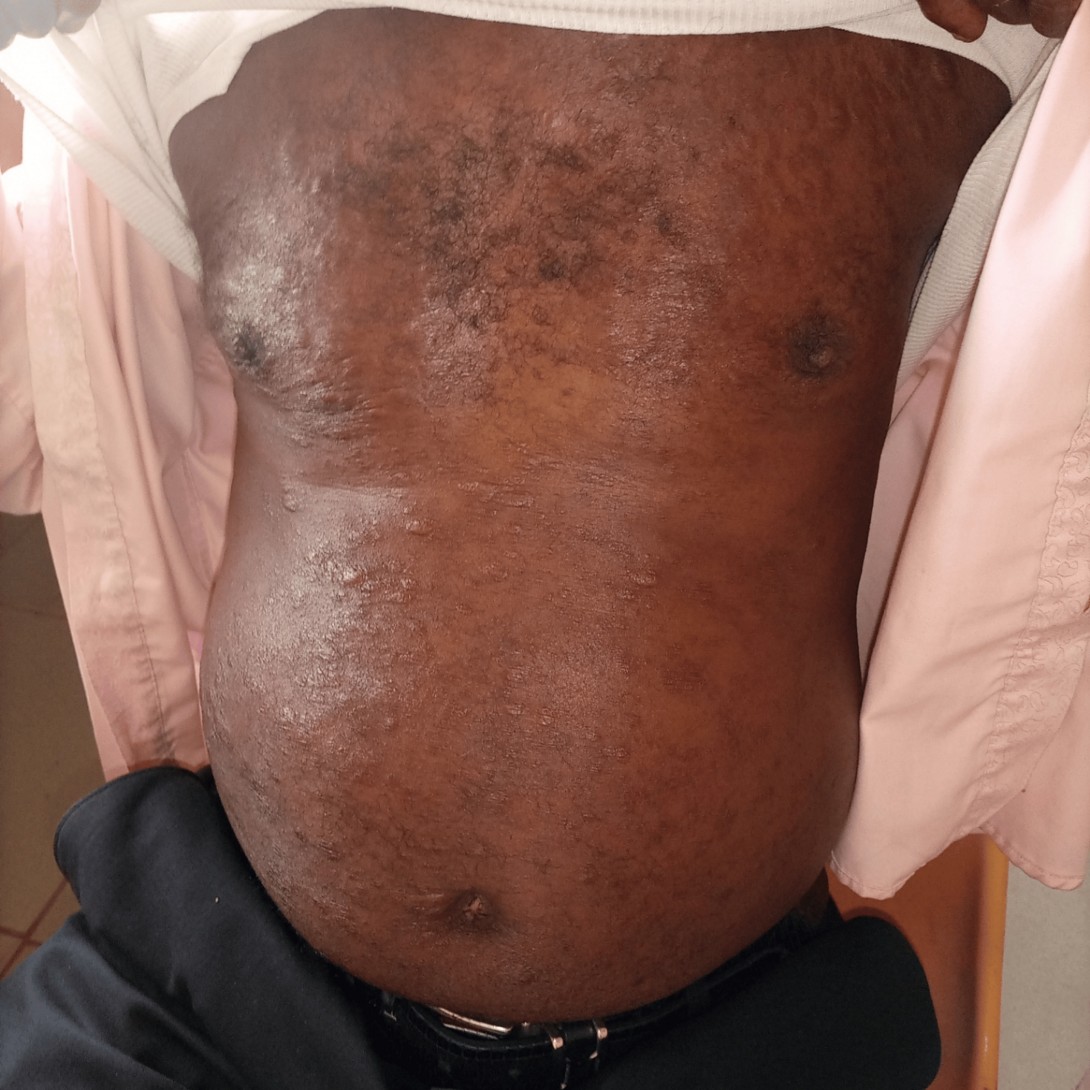
Clinical improvement following initiation of multidrug therapy, with reduction in nodular lesions and infiltration.

**Figure 6:**
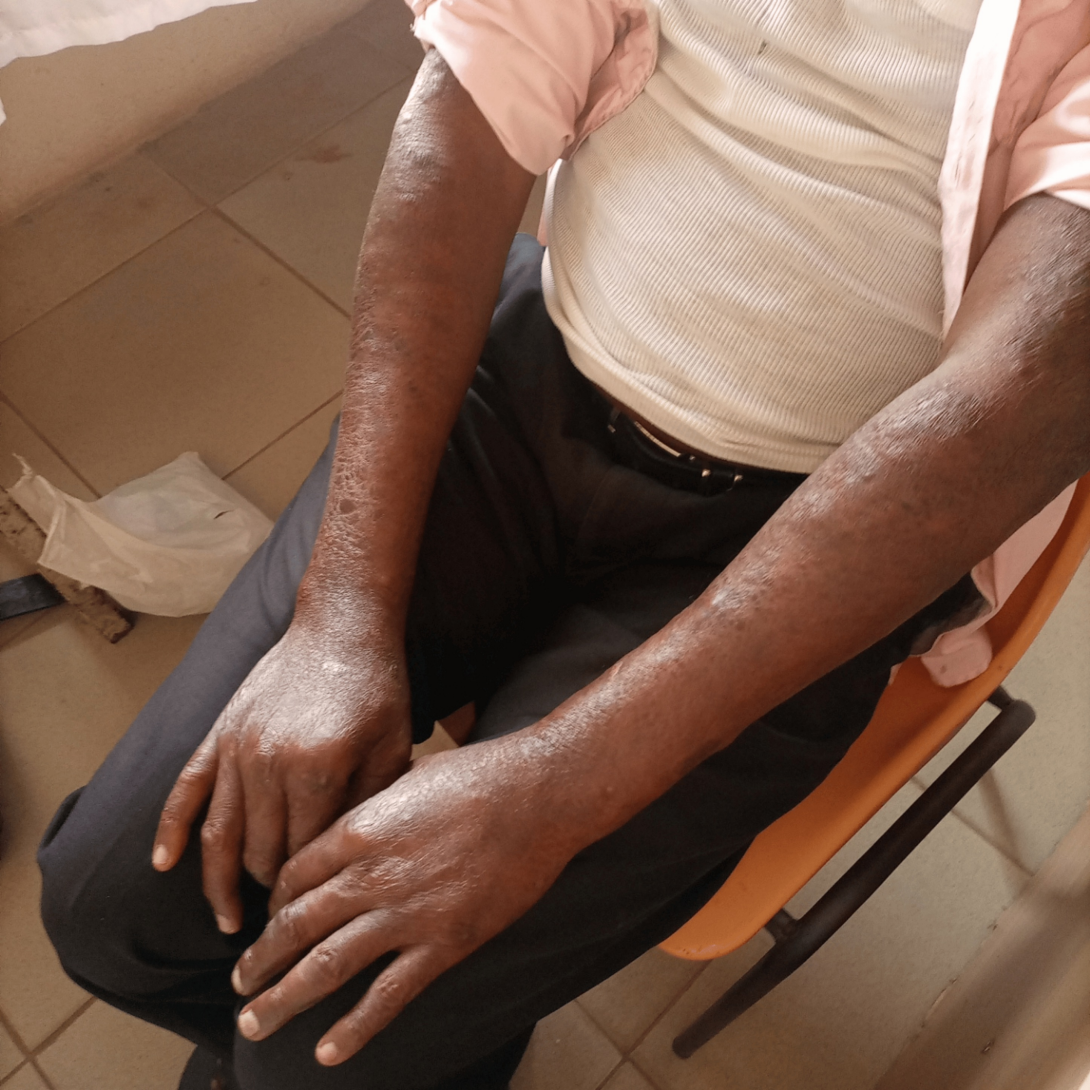
Clinical improvement following initiation of multidrug therapy, with reduction in nodular lesions and infiltration.

Following the final diagnosis, the patient was invited, counseled and was subsequently referred to the National TB Center Lagos where he was commenced on an MDT regimen comprising rifampicin, dapsone, and clofazimine with significant clinical improvement on follow-up (Figures 5, 6).

## Discussion

Leprosy was historically endemic worldwide until the late 19th century; however, it is now predominantly reported in tropical and subtropical regions. The disease is generally more prevalent in males than females, with a reported male-to-female ratio of approximately 2:1 [4]. Although leprosy can affect individuals of any age, children younger than 15 years constitute a considerable proportion of cases in developing countries such as Brazil [5]. Unlike tuberculosis, current evidence suggests that HIV co-infection does not significantly alter the clinical spectrum of leprosy or the distribution of lepromatous and tuberculoid forms [6].

Two bacterial species are known to cause leprosy: M. leprae and M. lepromatosis, a more recently identified species [7]. Clinically, infections caused by these organisms are largely indistinguishable [7,8]. Both are obligate intracellular, slow-growing, aerobic, Gram-positive pathogens [7]. Transmission is believed to occur primarily through the skin and nasal mucosa [9]. Evidence from systematic reviews suggests that bacilli may also be present in nasal secretions and, in patients with lepromatous leprosy, can be shed in large quantities [9]. Although the exact route of entry remains uncertain, current evidence supports both cutaneous and respiratory transmission pathways [9,3].

Determining the incubation period of leprosy is challenging due to its slow onset and the absence of reliable immunologic markers. According to the World Health Organization (WHO), up to 95% of individuals exposed to M. leprae do not develop clinical disease, suggesting that host immunity plays a major role in disease progression and control [3]. The incubation period is highly variable, ranging from approximately two to 20 years or longer [3]. Prolonged and close contact with an infected individual remains the most important risk factor for transmission and disease development, particularly in household settings, where exposure increases the likelihood of infection compared with the general population [9].

Additional risk factors for leprosy include residence in endemic regions such as India, Brazil, and Indonesia, as well as low socioeconomic status and conditions associated with impaired immunity, including malnutrition and other comorbid illnesses [2,3]. Although leprosy is not strongly influenced by HIV infection, current evidence suggests that HIV does not significantly increase susceptibility to the disease [6]. Genetic predisposition also plays an important role in disease susceptibility, with individuals carrying variants in immune-related genes such as NOD2, PARK2, PACRG, and lymphotoxin-α (LTA) being at increased risk [8]. Among these, a regulatory region on chromosome 6 affecting PARK2 and PACRG expression has been well documented in association studies [8]. The PARK2 gene encodes the parkin protein, which is involved in the ubiquitination pathway of protein degradation and has also been implicated in Parkinson’s disease [10,11].

In the Americas, the nine-banded armadillo (Dasypus novemcinctus) has been identified as a natural host and potential zoonotic reservoir for M. leprae [9]. However, in Nigeria, no confirmed non-human reservoirs have been reported [9]. The extent to which occupational exposure, such as work involving timber handling, may increase the risk of exposure to M. leprae remains uncertain and is not well established in the current literature. Further studies are required to clarify potential environmental or occupational risk factors in endemic settings.

After entering the body through the respiratory tract or via skin-to-skin contact, including exposure to secretions from lesions of infected individuals [3,9], M. leprae demonstrates a marked tropism for neural tissue and can invade Schwann cells. This interaction is largely mediated by toll-like receptors (TLRs) expressed on Schwann cells, which play an important role in host immune recognition [12]. Activation of these receptors by M. leprae has been suggested to contribute to cellular injury and subsequent nerve damage associated with the disease [12]. The organism has also been demonstrated in macrophages and other tissue cells, including muscle and endothelial cells, although the extent of involvement varies across studies [12].

Once inside Schwann cells or macrophages, the progression of infection depends largely on the host immune response [3,7,12]. M. leprae replicates slowly, with an estimated doubling time of approximately 12 to 14 days [7]. Following intracellular replication, the bacilli may spread to adjacent cells [7,12]. Infected individuals often remain asymptomatic during the prolonged incubation period of the disease [3]. As the bacterial burden increases, the host immune system responds through infiltration of lymphocytes and macrophages into affected tissues [7]. Clinical manifestations may subsequently develop, including peripheral nerve involvement associated with demyelination, impaired axonal conduction, sensory loss, and hypo-pigmented hairless skin lesions [7,13].

If left untreated, disease progression is largely determined by the strength of the host immune response [3]. Effective cell-mediated immunity (CMI) may control or eliminate the infection, resulting in spontaneous lesion resolution or the development of paucibacillary (PB) leprosy [7]. Conversely, impaired CMI permits uncontrolled bacterial proliferation, leading to multibacillary (MB) disease with more widespread systemic involvement [7]. In all forms of leprosy, M. leprae demonstrates a predilection for peripheral nerves, affecting both sensory and motor function through progressive nerve fiber damage [7,13]. Acute alterations in immune response, including those triggered by treatment initiation or immune restoration, may precipitate inflammatory episodes known as leprosy reactions, which can involve the skin, nerves, and other tissues [14].

Early in leprosy, small sensory and autonomic nerve fibers, as well as motor nerves, may become affected, resulting in localized hair loss, reduced sweating, and numbness [7,13,15]. Progressive nerve damage can subsequently lead to dry skin, muscle weakness or paralysis, and deformities such as claw hand, Z-thumb deformity, contractures, and digital shortening [13]. Skin fissures may also develop, predisposing affected individuals to secondary infections [7,13,15].

Lepromatous leprosy is characterized by widespread, bilaterally symmetrical lesions, including macules, nodules, plaques, and papules [8,13]. Advanced disease may result in leonine facies, madarosis involving the eyebrows and eyelashes, nasal collapse, ocular complications, lymphadenopathy, and testicular involvement with possible endocrine consequences [8]. Peripheral nerve involvement may be less clinically apparent in early disease despite heavy bacillary infiltration [7,12]. Histopathologically, lesions are characterized by poorly formed or absent granulomas, high bacillary loads, a Th2-predominant immune response, and impaired cell-mediated immunity [7,14].

Our 43-year-old Nigerian patient presented with extensive lepromatous lesions but preserved light touch sensation, an unusual finding in advanced multibacillary disease. Although sensory loss is a hallmark of leprosy, its onset and severity vary across the disease spectrum. In lepromatous leprosy, nerve involvement is typically diffuse and symmetrical, and clinical sensory impairment may be delayed despite significant bacillary infiltration [8,13,14]. Variability in the involvement of different sensory fiber types may further contribute to preserved sensation in early or atypical presentations [8,13,14]. This reflects a dissociation between bacillary burden and clinical nerve dysfunction in multibacillary disease, where extensive tissue infiltration does not always correlate with immediate sensory loss [8,13,14]. According to WHO guidelines, leprosy is primarily diagnosed on clinical grounds, with laboratory or histopathological findings serving as supportive evidence where available [3]. However, preserved sensation in lepromatous leprosy is uncommon and may complicate clinical recognition, potentially leading to diagnostic uncertainty. The patient was also evaluated for nasal symptoms initially suggestive of active rhinosinusitis or nasal polyposis; however, these findings may have reflected mucosal involvement related to leprosy-associated tissue changes. An initial clinical consideration of CTCL was later excluded following histopathological evaluation.

The distinction between CTCL and lepromatous leprosy can be challenging, particularly in cases with atypical clinical and histopathological features. In our patient, the initial histological finding of dense mononuclear infiltrates along the dermal-epidermal junction raised suspicion for CTCL, as this pattern may mimic epidermotropic lymphoid infiltration. However, subsequent evaluation demonstrated features more consistent with a reactive inflammatory process. Immunohistochemistry revealed a mixed inflammatory infiltrate composed of lymphocytes, histiocytes, and plasma cells. The presence of CD3 and CD45 positivity supported a T-cell-predominant inflammatory infiltrate, while CD68 highlighted a significant histiocytic component. Immunophenotypically, CTCL is typically characterized by a CD3+, CD4+ T-cell population with variable loss of pan-T-cell markers such as CD5 and CD7, whereas B-cell markers such as CD20 are generally absent, findings that help distinguish CTCL from reactive inflammatory infiltrates and other cutaneous lymphoid disorders [16-18]. In our patient, the absence of a consistent aberrant T-cell phenotype, including loss of CD5, together with the mixed inflammatory background, argued against a clonal lymphoproliferative process such as CTCL [16-18]. Overall, the immunophenotypic and histopathological findings were more consistent with an inflammatory dermatosis such as lepromatous leprosy rather than a primary CTCL.

In addition, inflammatory infiltrates involving peripheral nerve bundles are characteristic histopathological features of leprosy and are consistent with the predilection of M. leprae for peripheral nerves [7,12]. Diffuse dermal histiocytic infiltration with minimal granulomatous inflammation is more consistent with lepromatous leprosy, whereas tuberculoid forms are typically characterized by organized granulomatous inflammation composed of epithelioid histiocytes and Langhans giant cells [7]. These histopathological findings, together with the clinical presentation and immunohistochemical profile, supported the diagnosis of lepromatous leprosy. Although acid-fast staining, such as Fite staining, was not performed and represents a limitation of this case, the diagnosis was supported by the overall clinicopathologic and immunohistochemical correlation.

Two major classification systems are widely used in leprosy: the Ridley-Jopling classification and the WHO operational classification [3,19]. The Ridley-Jopling system classifies leprosy along an immunological spectrum based on the host response to M. leprae, ranging from tuberculoid leprosy at one pole to lepromatous leprosy at the opposite pole [19]. Between these extremes lie the borderline forms, including borderline tuberculoid, mid-borderline, and borderline lepromatous leprosy [19]. The position of a patient along this spectrum may change over time depending on disease progression and immune response [19]. Modern understanding of the immunological and histopathological features of this spectrum has been further elaborated in subsequent review studies [7,8]. In contrast, the WHO operational classification is primarily treatment-oriented and categorizes leprosy according to the number of skin lesions and bacteriological status [3]. Patients with a single skin lesion are classified as having single-lesion paucibacillary disease, those with five or fewer lesions are classified as paucibacillary leprosy, and those with more than five lesions are classified as multibacillary leprosy [3].

According to the WHO, leprosy may be diagnosed in an individual who has not completed appropriate therapy when at least one of the following cardinal features is present: hypopigmented or erythematous skin lesions with definite sensory loss, peripheral nerve involvement associated with nerve thickening and sensory impairment, or the demonstration of acid-fast bacilli on slit-skin smear examination [3]. WHO guidelines recommend that clinical findings be interpreted together with laboratory investigations when available [3]. Slit-skin smears and skin biopsies are commonly used to identify acid-fast bacilli, typically using the Fite stain, also known as the Wade-Fite or modified Ziehl-Neelsen technique [3]. Biopsy specimens should include the full thickness of the dermis and are preferably obtained from the active margin of a lesion to optimize diagnostic yield [3].

Several immunologic and molecular techniques may assist in the evaluation of leprosy, particularly in diagnostically challenging cases [20-23]. Polymerase chain reaction (PCR)-based methods have enabled the detection of M. leprae-specific DNA sequences in clinical specimens, including skin smears, nasal smears, tissue sections, blood samples, and biopsy material [20]. Serologic testing for antibodies against phenolic glycolipid-1 (PGL-1), a molecule specific to M. leprae, has demonstrated greater sensitivity in multibacillary and lepromatous forms of the disease than in paucibacillary forms [21-23]. Assessment of cell-mediated immunity using lymphocyte transformation or migration inhibition assays has shown that patients with lepromatous leprosy often exhibit impaired cellular immune responses to M. leprae, whereas individuals with tuberculoid disease typically retain stronger cell-mediated immunity [1,7,8]. The lepromin skin test, although not diagnostic for active infection, may be useful in evaluating host immune responsiveness to M. leprae. Positive reactions are more commonly observed in tuberculoid and borderline forms of leprosy, while patients with lepromatous disease frequently demonstrate little or no response [14]. Overall, both molecular and serologic methods tend to show lower sensitivity in paucibacillary disease because of the relatively low bacillary burden [20-23].

In 1982, the WHO introduced MDT for the treatment of leprosy using a combination of rifampicin, dapsone, and clofazimine [3]. Current WHO guidelines recommend six months of MDT for paucibacillary leprosy and 12 months for multibacillary disease [3]. The inclusion of clofazimine in the regimen for paucibacillary disease represents a modification of earlier WHO recommendations that used only rifampicin and dapsone for this group [3]. MDT has significantly improved treatment outcomes and reduced disease transmission worldwide, and patients are generally considered rapidly less infectious after initiation of appropriate therapy [2,3].

In cases of rifampicin-resistant leprosy, WHO recommends combination therapy using at least two second-line agents, such as clarithromycin, minocycline, or a quinolone, together with clofazimine [3]. For patients with additional fluoroquinolone resistance, treatment regimens may include clarithromycin, minocycline, and clofazimine during the intensive treatment phase, followed by prolonged continuation therapy with active agents and clofazimine [3]. Current recommendations for drug-resistant leprosy are largely based on expert consensus because evidence regarding alternative regimens remains limited [3].

Surgical intervention in leprosy is primarily aimed at correcting deformities and functional impairment resulting from peripheral nerve damage and motor paralysis. Common deformities include claw hand and thumb deformities caused by ulnar and median nerve dysfunction. Reconstructive procedures such as tendon transfer, arthrodesis, tenodesis, and Z-plasty may be used to improve hand function, correct contractures, and enhance joint stability in selected patients with irreversible nerve paralysis [24-26].

## Conclusions

Leprosy (Hansen’s disease) is a chronic infectious disease caused by M. leprae and M. lepromatosis, primarily affecting the skin, peripheral nerves, upper respiratory tract mucosa, and eyes. It is a curable condition, and early diagnosis and treatment are essential to prevent long-term disability.

We report a case of a 43-year-old Nigerian man, a timber contractor, who presented with progressive, non-pruritic papular lesions that initially involved the face and later became widespread, culminating in diffuse facial infiltration with leonine facies. Additional findings included nodular lesions on the extremities, some of which were ulcerated and secondarily infected, as well as infiltration of the earlobes with oil-drop nodules. These were associated with systemic weight loss.

A diagnosis of lepromatous leprosy was established based on clinicopathological correlation supported by histopathological and immunohistochemical findings. The patient was commenced on MDT (rifampicin, dapsone, and clofazimine), with progressive clinical improvement noted during follow-up.

A notable feature of this case was the preservation of light touch sensation despite extensive cutaneous involvement. While unusual in advanced lepromatous disease, this may reflect variability in the timing and extent of peripheral nerve involvement in multibacillary leprosy. This atypical presentation contributed to initial diagnostic consideration of CTCL, which was later excluded based on histopathological and immunohistochemical evaluation.

This case underscores the importance of maintaining a high index of suspicion for leprosy in endemic settings, even in the absence of classical sensory loss, and highlights the value of integrating clinical, histopathological, and immunohistochemical findings in establishing the diagnosis.
